# Structural Brain Network Alteration and its Correlation With Structural Impairments in Patients With Depression in *de novo* and Drug-Naïve Parkinson's Disease

**DOI:** 10.3389/fneur.2018.00608

**Published:** 2018-07-26

**Authors:** Lubin Gou, Wei Zhang, Chuanming Li, Xinlin Shi, Zhiming Zhou, Weijia Zhong, Ting Chen, Xiajia Wu, Chun Yang, Dajing Guo

**Affiliations:** Department of Radiology, The Second Affiliated Hospital of Chongqing Medical University, Chongqing, China

**Keywords:** depression, Parkinson's disease, structural brain network, diffusion tensor imaging, graph theory, tract-based spatial statistics

## Abstract

**Purpose:** Depression is common in Parkinson's disease (PD) and is correlated with the severity of motor deficits and quality of life. The present study aimed to investigate alterations in the structural brain network related to depression in Parkinson's disease (d-PD) and their correlations with structural impairments of white matter (WM).

**Materials and Methods:** Data were acquired from the Parkinson Progression Markers Initiative (PPMI) database. A total of 84 *de novo* and drug-naïve PD patients were screened and classified into two groups according to the 15-item Geriatric Depression Scale (GDS-15): d-PD (*n* = 28) and nondepression in PD (nd-PD, *n* = 56). Additionally, 37 healthy controls (HC) were screened. All subjects underwent DTI and 3D-T_1_WI on a 3.0 T MR scanner. Individual structural brain networks were constructed and analyses were performed using graph theory and network-based statistics (NBS) at both global and local levels. Differences in global topological properties were explored among the three groups. The association models between node and edge changes and the GDS-15 were constructed to detect regions that were specifically correlated with d-PD. Tract-based spatial statistics (TBSS) was used to detect structural impairments of WM between the d-PD and nd-PD groups. The correlations between altered global topological properties and structural impairments were analyzed in the d-PD group.

**Results:** The global efficiency and characteristic path length of the structural brain network were impaired in the d-PD group compared with those in the nd-PD and HC groups. Thirteen nodes and 1 subnetwork with 10 nodes and 12 edges specifically correlated with d-PD were detected. The left hippocampus, left parahippocampal, left lingual, left middle occipital, left inferior occipital, left fusiform, left middle temporal, and left inferior temporal regions were all involved in the results of node and edge analysis. No WM microstructural impairments were identified in the d-PD group.

**Conclusion:** Our study suggests that the integration of the structural brain network is impaired with disrupted connectivity of limbic system and visual system in the de novo and drug-naïve d-PD patients.The topological properties assessing integration of the structural brain network can serve as a potential objective neuroimaging marker for early diagnosis of d-PD.

## Introduction

Parkinson's disease (PD) is a progressive neurodegenerative disorder. Depression is one of the most common nonmotor symptoms of PD (1), which may present as the initial symptom, and can accelerate the progression of motor disablement and the decrease in quality of life in PD patients (2, 3). However, given the lack of sufficient attention to the presence and severity of depression in Parkinson's disease (d-PD), most d-PD patients do not receive timely and appropriate treatment and are faced with the risks of decreased motor function and quality of life (3). It is therefore essential to reveal the pathophysiological mechanism of d-PD for the early diagnosis and precise treatment.

The white matter (WM) is the anatomical basis of brain information or nerve impulse transfer. Revealing how alteration of the WM is related to d-PD can help uncover the neurological mechanism of depressive symptoms in PD. Diffusion tensor imaging (DTI) provides noninvasive methods to explore WM changes and to elucidate the neural substrates of d-PD *in vivo*. To date, only a few DTI studies have attempted to identify regional defects in the WM in d-PD patients. Previous studies have shown that the WM microstructure was impaired mainly in the uncinate fasciculus and in some long contact fibers of d-PD patients (4, 5). However, the dopaminergic, noradrenergic and serotonergic systems have all been discovered to be related to the pathology of d-PD in sporadic brain regions, such as the striatum, limbic system, and thalamus (6). These complex alterations involving multiple neurotransmitter systems suggest that depressive symptoms do not simply result from regional deficits in PD; instead, disordered interconnections of brain regions may be due to d-PD.

Compared with approaches analyzing localized brain changes, understanding the brain as the network of interconnected regions has been highlighted (7). The structural brain network based on DTI is a novel approach to describe the overall organization of brain WM. Using graph theory, information transmission within groups of densely interconnected regions and specialized information exchange between distributed groups, also known as the segregation and integration of the structural brain network, and the importance (centrality) of a brain region can be assessed by the topological properties. The edges with altered connective strength can also be detected using network-based statistics (NBS). In previous studies, PD has been considered to be a disconnection syndrome of the structural brain network with reduced integration and segregation compared to healthy controls (8, 9). And decreased connective strength was identified in both motor and nonmotor circuits in PD patients using NBS (10). However, how depressive symptoms are related to alterations of the structural brain network in PD patients remains unclear.

Therefore, in present study, we applied the graph theory and NBS approach to analyze the structural brain network in de novo and drug-naïve PD patients with and without depression and healthy controls, which may allow the effect of d-PD in the structural brain network to emerge. Since the structural brain network was constructed based on the macro fibers, analyzing microstructural alterations of the WM and their interactions with network impairment together can provide further insights into the neural substrate of d-PD. We further explored impairment of WM integrity in d-PD patients using tract-based spatial statistics (TBSS) and analyzed its correlation with the global topological properties of the structural brain network. We hypothesized that (1) integration of the structural brain network was impaired and affected extensive brain regions in d-PD patients, and (2) there were some impairments of WM integrity that correlated with impairments of structural brain network.

## Materials and methods

### Participants

The data used in this study were downloaded from the Parkinson Progression Markers Initiative (PPMI) database via a standard application process (http://www.ppmi-info.org). The PPMI is an international multicenter study designed to identify biomarkers of PD progression through clinical, imaging, and biospecimen data. Ethics approval of this study was obtained from the Institutional Review Board or Independent Ethics Committee of each respective center, and written informed consent was obtained from all participants in the PPMI study.

Note that disease duration and anti-Parkinsonism may affect the brain network organization to some degree (11). We screened 84 PD patients at baseline and 37 healthy controls (HCs) in the PPMI database. The inclusion criteria of PD mainly included the following: (1) motor symptoms, including asymmetric resting tremor or rigidity, or two of resting tremor, rigidity and bradykinesia; (2) dopamine transporter (DAT) imaging revealing a DAT deficit; (3) diagnosis within 2 years; (4) no medication or surgery for PD; (5) Hoehn and Yahr (H&Y) stage 1 or 2; and (6) availability of DTI and 3D-T_1_WI images without artifacts, obvious head motion and distortion. The exclusion criteria mainly included the following: (1) atypical PD syndromes due to drugs, metabolic disorders, encephalitis, or degenerative diseases; (2) clinical diagnosis of dementia; and (3) MRI scan with evidence of clinically significant neurological disorder. HCs were required to have normal DATs, no significant neurological disorders and 15-item Geriatric Depression Scale (GDS-15) scores lower than 5. Details regarding the inclusion and exclusion criteria are presented in the supplementary materials.

The motor function of PD patients was evaluated using the Movement Disorder Society Unified Parkinson's Disease Rating Scale III (MDS-UPDRS-III). Data pertaining to the side of onset and motor features in PD were also collected. Data from the Montreal Cognitive Assessment (MoCA) and education years were assessed to control for cognitive factors in all PD patients and HCs.

Depression severity was assessed by the GDS-15 in all PD patients. The GDS-15 is a brief self-report measure focusing on the psychological aspects (e.g., hopelessness) and social consequences of depression. The GDS-15 is a popular tool for screening d-PD with high specificity (12). According to GDS-15 scores, the PD patients were divided into two study groups: 28 d-PD patients with GDS-15 scores greater than or equal to 5 and 56 nd-PD patients with scores lower than 5 (13).

### MRI protocol

This data set was acquired using a standardized protocol (details can be found in the MRI technical operation manual at the appropriate website) on Tim Trio 3 Tesla Siemens scanners (Siemens, Erlangen, Germany). The protocol includes the following parameters: (1) DTI sequence: 72 axial slices, echo time (TE) = 88 ms, repetition time (TR) = 500–9,000 ms, flip angle = 90°, voxel size: 2.0 × 2.0 × 2.0 mm^3^, acquisition matrix = 1044 × 1044; 64 diffusion-sensitive gradient directions at *b* = 1,000 s/mm^2^ and one diffusion-unweighted (b0) image; (2) 3D-T_1_WI sequence: 176 sagittal slices, TE = 2.98 ms, TR = 2300 ms, flip angle = 9°, voxel size: 1.0 × 1.0 × 1.0 mm^3^, acquisition matrix = 240 × 256.

### DTI preprocessing and tractography

All DTI image preprocessing was implemented using a pipeline toolbox in MATLAB, PANDA (14). Briefly, stripping nonbrain tissue and estimating the brain mask were first performed using the brain extraction tool. Then, the eddy-current effect and simple head motion were corrected. The diffusion tensor (DT) matrix was calculated on a voxelwise basis to obtain fractional anisotropy (FA) and mean diffusivity (MD) maps.

Whole-brain WM fiber reconstruction was performed with PANDA using the Diffusion Toolkit. Deterministic tractography was conducted using the Fiber Assignment by Continuous Tracking (FACT) algorithm and was terminated if a voxel with FA less than 0.2 or a curvature angle greater than 45° was encountered.

### Network construction

Node and edge definitions: To ensure the accuracy of brain parcellation, structural images were reoriented to the Montreal Neurological Institute (MNI) template manually using Statistical Parametric Mapping 12 (SPM12; Institute of Neurology, London, UK). The cortical and subcortical regions (without the cerebellum) were segmented as nodes by PANDA using the automated anatomical labeling (AAL) atlas that includes a total of 90 cortical and sub-cortical regions (45 for each hemisphere). Using an affine transformation, individual FA maps were co-registered to the corresponding T1-weighted structural images, which were then non-linearly registered to the MNI-ICBM152_2 mm template. The results of this two-step registration were used to calculate the combined inverse warp that allowed co-registration of the AAL atlas to subject-specific FA maps to generate the individual AAL atlas. Since the AAL atlas is coarse, we multiply the gray matter (GM) mask to avoid containing WM in the individual atlas. The edges were represented by the WM tracts obtained by deterministic tractography linking each pair of nodes.

Network construction: PANDA was used to obtain a connectivity matrix from the defined regions in the individual AAL atlas and tractography for network construction. To avoid the presence of spurious fibers, we set all connections with fewer than three fibers to zero. The number of fibers (FN) was used as a measure of edge connective strength, namely, the weight of an edge. The individual brain structural FN-weighted network was calculated for each subject included in the study.

### Structural brain network analyses

Graph theory and NBS were applied to analyze the structural brain network. To investigate the integration of the structural brain network, we explored global efficiency and characteristic path length. Segregation was assessed by the clustering coefficient, local efficiency, and modularity. Small-world organization was assessed by the normalized characteristic path length, the normalized clustering coefficient and small-worldness.

The local topological properties (degree, betweenness, nodal global efficiency and nodal local efficiency) were obtained to assess the centrality of a node. All calculations of topological properties were performed using the MATLAB toolbox of GRETNA (15).

The NBS approach was utilized to detect abnormalities of edge connective strength, and the family-wise error rate (FWER) was controlled (16). This process consists of four steps. First, independently test the hypothesis of interest at every connection in the network. Second, choose a primary test statistic threshold. Third, identify subnetworks among the set of supra-threshold connections using a breadth or depth search. Finally, compute the FWER-corrected *p*-value for each component using permutation testing.

To identify the specific nodes and edges associated with d-PD, we constructed a correlation model of node and edge alterations and depression (assessed by GDS-15 scores). We screened the nodes exhibiting three or more topological properties correlated withGDS-15 scores and the largest subnetwork in the NBS results in the analyses. All results were visualized using BrainNet Viewer (17).

### WM structural analysis

To further explore the correlations between global topological properties and WM microstructural impairments in d-PD, FA, and MD values were used to measure microstructural changes. The comparison of FA and MD data between the groups and the correlations between FA/MD and GDS-15 scores were implemented at the voxel level using TBSS (http://fsl.fmrib.ox.ac.uk/fsl/fslwiki/TBSS). TBSS processing involves a few simple steps: first, apply nonlinear registration of all FA images into standard space; second, create the mean FA image and skeletonize it; and, finally, project all subject's FA data onto the mean FA skeleton with FA value greater than 0.2.

### Statistical analyses

To calculate significant differences for demographic and clinical characteristics, ANOVA was used for age, MoCA scores and education years, and a pairwise post-hoc test was subsequently used to identify significant main group differences with correction by the Bonferroni method. χ^2^ tests were used to analyze sex among the three groups. Two independent-samples *t*-tests were used for GDS-15 and UPDRS-III scores, and χ^2^ tests were used to analyze H&Y stage and the side of onset between the d-PD and nd-PD groups. *P* < 0.05 was considered significant after correction.

All the structural brain network analyses were based on a general linear model (GLM), with age and UPDRS-III scores as covariates. To detect impairment of the whole structural brain network, global topological properties were compared among the d-PD, nd-PD, and HC groups. A pairwise post-hoc test was subsequently used to identify significant main group differences with Bonferroni correction for multiple comparisons. *P* < 0.05 was considered significant after correction.

Correlations between nodal topological property alterations and GDS-15 scores were calculated by regression analysis with Bonferroni correction for multiple comparisons. *P* < 0.01 was considered significant after correction.

Both positive and negative correlations between edge connective strength (defined by the FN) and GDS-15 scores in PD patients were explored using the nonparametric permutation statistic (5,000 permutations; *P* < 0.001) with NBS correction. A range of primary test statistic thresholds was tested to determine the threshold value with the most robust result. The primary test statistic threshold was set to 2.6 according to the test results (details are shown in the supplementary materials).

Receiver operating characteristic (ROC) curve analysis was used to quantify the ability of significant impaired topological properties in d-PD to enable discrimination between d-PD and nd-PD patients, with the significance level set to *P* < 0.05.

The randomize tool was used in TBSS based on the nonparametric permutation statistic: 5,000 permutations; *P* < 0.05. Multiple comparisons were corrected at the cluster level using the threshold-free cluster enhancement (TFCE) and FWER option, and age and UPDRS-III scores were set as covariates.

Spearman correlation coefficients were calculated between the global topological properties and DTI indices (the mean FA and MD values of one region) in d-PD group. *P* < 0.05 was considered significant.

## Results

### Demographic and clinical characteristics

Table 1 summarizes the demographic and clinical characteristics of the d-PD patients, nd-PD patients and HCs. No significant differences were found in sex, age, MoCA scores, and education years among the three groups. No significant differences were found in UPDRS-III scores, H&Y stage and the side of onset between the d-PD and nd-PD groups. GDS-15 scores were statistically significantly different between the d-PD and nd-PD groups.

**Table 1:** Demographic and clinical characteristics of the d-PD patients, nd-PD patients and HCs.

**Characteristic**	**d-PD(*n* = 28)**	**nd-PD(*n* = 56)**	**HC(*n* = 37)**	***P-*value for the three groups**	***P-*value for d-PD vs. nd-PD**	***P-*value for HC vs. d-PD**	***P-*value for HC vs. nd-PD**
	**Mean ± SD**	**Mean ± SD**	**Mean ± SD**				
**Sex (male/female)**	17/11	36/20	21/16	0.708			
**Age**	61.43 ± 10.06	63.97 ± 8.31	60.35 ± 11.70	0.259	0.952	>0.999	0.338
**MoCA**	27.71 ± 1.76	27.7 ± 2.05	28.24 ± 1.23	0.209	>0.999	0.710	0.251
**Education (year)**	15.36 ± 3.37	15.93 ± 3.02	15.62 ± 2.92	0.673	>0.999	>0.999	>0.999
**GDS-15**	7.07 ± 2.35	1.39 ± 1.29			0.000^*^		
5–7	18						
8–11	8						
12–15	2						
**MDS-UPDRS-III**	25.32 ± 9.08	22.419.51			0.473		
**H&Y Stage (1/2)**	6/22	21/35			0.813		
**Side of onset**					>0.999		
Left	11	24					
Right	16	31					
Symmetric	1	1					
**Motor feature**							
Resting tremor	20	45					
Rigidity	21	47					
Bradykinesia	25	48					
Postural instability	7	1					

* *Statistical significance; H & Y, Hoehn & Yahr staging; MoCA, Montreal Cognitive Assessment; GDS-15, Geriatric Depression Scale-Short; MDS-UPDRS-III, Movement Disorder Society Unified Parkinson's Disease Rating Scale III*.

### Global topological properties analysis

At the global level, global efficiency was significantly decreased and characteristic path length was significantly increased in the d-PD group compared with those in the nd-PD and HC groups. Local efficiency was decreased in the d-PD group compared with that in the nd-PD group. The normalized characteristic path length was decreased and small-worldness was increased in the nd-PD patients compared with those in the HCs (Table 2).

**Table 2:** Results of global topological properties.

**Global topological properties**	**d-PD(*n* = 28)**	**nd-PD(*n* = 56)**	**HC(*n* = 37)**	***P-*value for the three groups**	***P-*value for d-PD vs. nd-PD**	***P-*value for HC vs. d-PD**	***P-*value for HC vs. nd-PD**
	**Mean ± SD**	**Mean ± SD**	**Mean ± SD**				
Global efficiency	35.13 ± 21.53	50.14 ± 12.25	54.29 ± 16.68	<0.001^*^	<0.001^*^	0.018^*^	>0.999
Characteristic path length	0.07 ± 0.09	0.02 ± 0.01	0.02 ± 0.01	<0.001^*^	<0.001^*^	<0.001^*^	0.892
Local efficiency	70.29 ± 42.94	99.96 ± 19.92	91.13 ± 16.36	<0.001^*^	<0.001^*^	0.130	0.999
Clustering coefficient	0.033 ± 0.009	0.032 ± 0.009	0.028 ± 0.011	0.414			
Modularity	0.67 ± 0.03	0.68 ± 0.02	0.64 ± 0.06	0.095			
Normalized clustering coefficient	6.57 ± 1.04	6.43 ± 1.05	5.08 ± 1.82	0.060			
Normalized characteristic path length	0.77 ± 0.05	0.76 ± 0.04	0.81 ± 0.08	0.012^*^	>0.999	0.065	0.009^*^
Small-worldness	8.58 ± 1.72	8.51 ± 1.74	6.45 ± 2.79	0.044^*^	>0.999	0.069	0.044^*^

* *Statistical significance*.

In the ROC analysis, global efficiency enabled differentiation between d-PD and nd-PD patients, with an area under the ROC curve (AUC) of 0.693, *P* = 0.002. Characteristic path length enabled differentiation between d-PD and nd-PD patients, with an AUC of 0.694, *P* = 0.002.

### Nodes and edges correlated with D-PD

**Figure 1:**
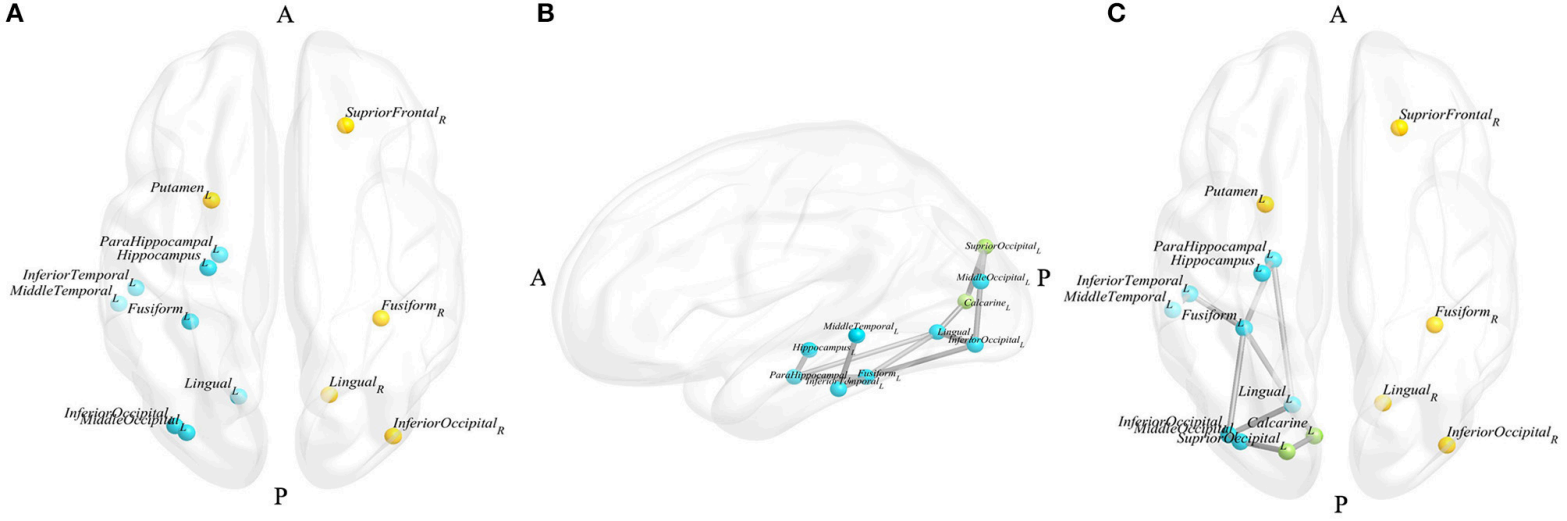
Blue nodes represent the nodes that appeared in the results of the node and edge analyses, yellow nodes represent the nodes that only appeared in the results of the node analysis, and green nodes represent the nodes that only appeared in the results of the edge analysis. **(A)** Nodes significantly correlated with d-PD; **(B)** the largest subnetwork significantly correlated with d-PD in the left hemisphere; **(C)** nodes and edges significantly correlated with d-PD. (A, anterior, P, posterior, L, left, R, right).

All the node topological properties assessed in the present study were negatively related with GDS-15 scores. Thirteen nodes were found to be significantly and specifically associated with depressive symptoms in PD patients, which involved the right superior frontal, left hippocampus, left parahippocampal, bilateral lingual, left middle occipital, bilateral inferior occipital, bilateral fusiform, left putamen, left middle temporal, and left inferior temporal regions (Figure 1A).

The largest subnetwork, which the edge connective strengths were negatively correlated with GDS-15 scores, including the left hippocampus, left parahippocampal, left calcarine, left lingual, left superior occipital, left middle occipital, left inferior occipital, left fusiform, left middle temporal, left inferior temporal gyrus and 12 edges connecting these nodes (the details are shown presented in Table 3, Figure 1B). No subnetworks were detected with edge strength associated with GDS-15 scores positively.

**Table 3:** The components of the largest subnetwork with a negative correlation with GDS-15 scores identified by NBS.

Edge^*^	**Node 1**	**Node 2**	***P***
1	Hippocampus_L	ParaHippocampal_L.	<0.001
2	ParaHippocampal_L	Lingual_L.	<0.001
3	Calcarine_L	Lingual_L.	<0.001
4	Calcarine_L	Occipital_Sup_L.	<0.001
5	Occipital_Sup_L	Occipital_Mid_L.	<0.001
6	Lingual_L	Occipital_Inf_L.	<0.001
7	Occipital_Mid_L	Occipital_Inf_L.	<0.001
8	ParaHippocampal_L	Fusiform_L.	<0.001
9	Lingual_L	Fusiform_L.	<0.001
10	Occipital_Inf_L	Fusiform_L.	<0.001
11	Fusiform_L	Temporal_Inf_L.	<0.001
12	Temporal_Mid_L	Temporal_Inf_L.	<0.001

* *The edge refers to the connection between node 1 and node 2. (L, left, R, right)*.

The nodes in the limbic system and the occipital and temporal lobes, including the left hippocampus, left parahippocampal, left lingual, left middle occipital, left inferior occipital, left fusiform, left middle temporal and left inferior temporal, were involved in the results of both node and edge analyses (Figure 1C).

### TBSS results and relationship with global topological properties

After TFCE correction, reduced FA was identified in forceps major, left uncinate fasciculus, left superior longitudinal fasciculus, bilateral corticospinal tract and arcuate fibers in the bilateral frontal and right occipital regions. The pattern of increased MD covered several regions, including the genu of the corpus callosum, the body and column of the fornix, the left crus of the fornix, bilateral corticospinal tract and arcuate fibers in the left amygdala, left insula, bilateral frontal, bilateral parietal, bilateral temporal and right occipital regions (*P* < 0.05, TFCE corrected). No significant increased FA or reduced MD was identified in the d-PD group compared with the nd-PD group (*P* > 0.05, TFCE corrected). After TFCE and FWE correction, no significant differences were found in the FA and MD maps of the whole WM skeleton between the d-PD and nd-PD groups. No significant correlations were found between the FA and MD values and GDS-15 scores in PD patients after TFCE and FWE correction.

## Discussion

In the present study of de novo and drug-naïve PD patients, we compared global topological properties among d-PD, nd-PD and HC groups and detected brain regions that were specifically associated with d-PD based on the local topological properties and edge connective strength. We also explored whole-brain impairments in d-PD patients of WM integrity using TBSS. Our study revealed the following main findings. First, compared to nd-PD patients and HC, d-PD patients showed impaired global efficiency and characteristic path length of the structural brain network, and global efficiency and characteristic path length were valuable in diagnosis of d-PD. Second, the limbic system, occipital and temporal lobes involved in the impairments of structural brain network were correlated with depressive symptom in PD patients. Third, no significant differences of WM microstructure were found between d-PD and nd-PD group.

The modality of whole-brain information flow can be conceptualized as the structural brain network. Increased characteristic path length and reduced global efficiency, as observed in the d-PD patients in our study, reflect breakdown of network integration. Similarly, one functional MRI (fMRI) study revealed an increased characteristic path length of the functional network in d-PD patients compared with that in healthy individuals in specific frequencies of signal oscillation (18). Integration of the structural brain network refers to specific information transmission and exchange between distributed areas (19). Impairments of integration suggest the presence of reduced efficiency and extra costs of information transmission between functional areas in d-PD. One of the significant somatic symptoms of d-PD is psychomotor retardation, which manifests as pervasive slowing-down of thought and reduced physical movements (20). Altered information exchange among the whole brain may result in this asystematic manifestation in d-PD.

Corresponding to the impaired integration of the structural brain network at the global level, the nodes associated with d-PD were distributed mainly in the limbic system and visual system, and the edges between the limbic system and visual system were also found to be associated with d-PD in our study. On top of these, the severity of depression was negatively associated with the node centrality and edge connective strength. The more important a node is, the more vulnerable the node is to be insulted (19). The decreased centrality reflected the impairments of node, which would lead to the longer path of information transfer. The reduced edge strength would lead to the inefficiency of information transfer. As such, impairments of the structural brain network of d-PD made the information between limbic system and visual system difficult to spread.

The parahippocampal and hippocampus as parts of the limbic system participate in emotional perception and reward behavior (21, 22). Several morphological studies have shown that the cortex thickness of the parahippocampal (23) and the volume of the hippocampus (24) were negatively related to depression severity in PD patients, and one fMRI study showed that the functional connectivity of the left parahippocampal was decreased in d-PD patients compared to that in HCs (25). Consistent with previous studies, our results also showed that the parahippocampal and hippocampus were associated with depression severity in PD patients. Therefore, our study further suggests that the limbic system play a critical role in the presence of d-PD.

In our results, several nodes in occipital and visual temporal (middle temporal and inferior temporal) were associated with d-PD. The occipital lobe and visual temporal are both important in the visual system. One study showed that depression was correlated with impaired color vision in PD patients through clinical observation and inferred that the visual system is crucial in the depressive pathology in PD patients (26). Some fMRI studies have shown that the value of regional homogeneity in the occipital lobe was decreased (27) and the synchrony of interhemispheric resting-state functional connectivity was impaired in the occipital lobe in d-PD patients (28).

The reduction of connectivity strength between limbic system and visual system may result in deficits of information transmission from the visual system to the reward circuit, and the reward circuit cannot be completely active. Subsequently, depressed mood, loss of pleasure (anhedonia), and feelings of worthless or guilt, which are the core symptoms of d-PD, can emerge in PD patients. Similarly, a previous study in major depressive disorder inferred that visual sensory abnormalities may cause loss of interest in daily activities and an inability to experience pleasure, and normal sensory stimulations are no longer present to activate the reward circuit (29). However, visual disturbance is also an independent sensory symptom in PD and is not the same as the visual dysfunction included among the depressive symptoms of major depressive disorder. The causal relationship between depression and visual function should be further studied in PD patients.

A previous study indicated that a relatively hypoactive left hemisphere is correlated with depression (30). A similar finding was demonstrated in our study, and most of the nodes and edges that were found to be correlated with d-PD were located in the left hemisphere, suggesting the asymmetry of the structural brain network in d-PD patients.

Contrary to our hypothesis, the WM microstructure was intact in d-PD patients in the present study. Two DTI studies showed that the integrity of long contact fibers was impaired in d-PD patients, but the d-PD patients were exposed to anti-parkinsonism medicine in these studies (4, 5). Consistent with our results, another study in PD patients with mild depression showed that the limbic-frontal fibers were intact (31). Therefore, our TBSS result can be explained by the fact that all PD patients were drug-naïve and most d-PD patients exhibited minor depression in the present study. Considering the network results, we can infer that impaired integration of the structural brain network emerges earlier than disruption of the microstructure in early-stage d-PD patients. In addition, the results of the ROC curve analysis showed that global efficiency and characteristic path length can distinguish d-PD patients from nd-PD patients. Therefore, our results indicate that the integration of the structural brain network can serve as an objective indicator for early diagnosis of d-PD.

Some limitations of our study should be noted. First, we analyzed the structural brain network based on DTI. It is generally known that the ability to resolve crossing fibers is limited in present DTI processing. However, the DT model is robust to noise and is widely used in WM structural analyses (32). Second, the sample size was relatively small, especially in the d-PD group. Hence, a greater number of d-PD patients should be included in future studies. Third, the d-PD patients mainly exhibited mild depression according to the corresponding GDS-15 category in the present study, and this uneven distribution may lead to inaccurate results. Fourth, a previous study revealed that PD patients with mild cognitive impairment exhibited abnormal structural brain networks (11). Therefore, we controlled for cognitive factors and excluded their interference in the present study. However, other nonmotor features that may affect the structural brain network, such as visual hallucinations, were not considered. Future studies should include these factors as control variables.

In conclusion, our study explored the structural brain network of de novo and drug-naïve d-PD patients based on DTI. Our results indicate that integration of the structural brain network is disrupted and mainly involves limbic system and visual system in *de novo* and drug-naïve d-PD patients. This impairment emerges earlier than WM microstructural deficits and may serve as a potential neuroimaging marker for early diagnosis of d-PD.

## Ethics statement

The study was approved by the Institutional Review Board or Independent Ethics Committee of all participating sites in Europe, including Attikon University Hospital (Greece), Hospital Clinic de Barcelona and Hospital Universitario Donostia (Spain), Innsbruck University (Austria), Paracelsus-Elena Clinic Kassel/University of Marburg (Germany), Imperial College London (UK), Pitié-Salpêtrière Hospital (France), University of Salerno (Italy), and in the USA, including Emory University, Johns Hopkins University, University of Alabama at Birmingham, PD and Movement Disorders Center of Boca Raton, Boston University, Northwestern University, University of Cincinnati, Cleveland Clinic Foundation, Baylor College of Medicine, Institute for Neurodegenerative Disorders, Columbia University Medical Center, Beth Israel Medical Center, University of Pennsylvania, Oregon Health and Science University, University of Rochester, University of California at San Diego, and University of California, San Francisco.

## Author contributions

LG and WZ equally contributed to this work and should be considered co-first authors. LG and WZ designed the study. LG, WZ, TC, XS, CY, XW, WJZ, and ZZ processed and analyzed the data. LG wrote the article. DG, CL, and WZ contributed to revision of the article.

### Conflict of interest statement

The authors declare that the research was conducted in the absence of any commercial or financial relationships that could be construed as a potential conflict of interest.
