# Supplementary material for: Structural Brain Network Alteration and its Correlation With Structural Impairments in Patients With Depression in de novo and Drug-Naïve Parkinson's Disease
Source: Front Neurol. 2018 Jul 26;9:608. doi: 10.3389/fneur.2018.00608 (PMC6070599; doi:10.3389/fneur.2018.00608)
Supplement: Supplementary file 1 [file Table_1.DOCX]

Supplementary Materials

The details of inclusion and exclusion criteria for all subjects

1. Inclusion Criteria (Parkinson Disease Subjects)
1.1. Patients must have at least two of the following: resting tremor, bradykinesia, rigidity (must have either resting tremor or bradykinesia); OR either asymmetric resting tremor or asymmetric bradykinesia.
1.2. A diagnosis of Parkinson disease for 2 years or less at Screening.
1.3. Hoehn and Yahr stage I or II at Baseline.

1.4. Not expected to require PD medication within at least 6 months from Baseline.
1.5. Male or female age 30 years or older at time of PD diagnosis.
1.6. Confirmation from imaging that screening dopamine transporter SPECT scan is consistent with dopamine transporter deficit (or for sites where DaTSCANTM is not available that VMAT-2 PET scan is consistent with VMAT deficit).
1.7. Ability to provide written informed consent in accordance with Good Clinical Practice (GCP), International Conference on Harmonization (ICH), and local regulations.
1.8. Willing and able to comply with scheduled visits, required study procedures and laboratory tests.
1.9. Women may not be pregnant, lactating or planning pregnancy during the course of the study.
• Includes a negative urine pregnancy test on day of Screening scan prior to injection (DaTSCANTM and/or 18F-AV-133).

• Includes a negative serum pregnancy test prior to Screening scan injection (18F-AV-133 only).
1.10. Women participating in VMAT-2 PET imaging must be of nonchildbearing potential **or** be using a highly effective method of birth control 14 days prior to until at least 24 hours after injection of 18F-AV-133).
• Non-child bearing potential is defined as a female that must be either postmenopausal (no menses for at least 12 months prior to Screening) or surgically sterile (bilateral tubal ligation, bilateral oophorectomy or hysterectomy).
• Highly effective method of birth control is defined as practicing at least one of the following: A birth control method that results in a less than 1% per year failure rate when used consistently and correctly, such as oral contraceptives for at least 3 months prior to injection, an intrauterine device (IUD) for at least 2 months prior to injection, or barrier methods, e.g., diaphragm or combination condom and spermicide. Periodic abstinence (e.g. calendar, ovulation, symptothermal, post-ovulation methods) is not acceptable.
**2. Exclusion Criteria (Parkinson Disease Subjects)**2.1. Atypical PD syndromes due to either drugs (e.g., metoclopramide, flunarizine, neuroleptics) or metabolic disorders (e.g., Wilson’s disease), encephalitis, or degenerative diseases (e.g., progressive supranuclear palsy).
2.2. Currently taking levodopa, dopamine agonists, MAO-B inhibitors (e.g., selegiline, rasagiline), amantadine or other PD medication.
2.3. Has taken levodopa, dopamine agonists, MAO-B inhibitors or amantadine within 60 days of Baseline.
2.4. Has taken levodopa or dopamine agonists prior to Baseline for more than a total of 60 days.
2.5. A clinical diagnosis of dementia63 as determined by the investigator (Appendix 1).
2.6. Received any of the following drugs that might interfere with dopamine transporter SPECT imaging: Neuroleptics, metoclopramide, alpha methyldopa, methylphenidate, reserpine, or amphetamine derivative, within 6 months of Screening.
2.7. Subjects participating in VMAT-2 PET imaging have received any of the following medications that might interfere with 18F-AV-133 PET imaging: neuroleptics, metoclopramide, alpha methyldopa, methylphenidate, reserpine, or amphetamine derivative, within 2 weeks prior to the Screening 18F-AV-133 injection.
2.8. Current treatment with anticoagulants (e.g., coumadin, heparin) that might preclude safe completion of the lumbar puncture.
2.9. Condition that precludes the safe performance of routine lumbar puncture, such as prohibitive lumbar spinal disease, bleeding diathesis, or clinically significant coagulopathy or thrombocytopenia.

2.10. Any other medical or psychiatric condition or lab abnormality, which in the opinion of the investigator might preclude participation.
2.11. Use of investigational drugs or devices within 60 days prior to Baseline (dietary supplements taken outside of a clinical trial are not exclusionary, e.g., coenzyme Q10).
2.12. Previously obtained MRI scan with evidence of clinically significant neurological disorder (in the opinion of the Investigator).
**3. Inclusion Criteria (Healthy Control Subjects)**3.1. Male or female age 30 years or older at Screening.
3.2. Ability to provide written informed consent in accordance with Good Clinical Practice (GCP), International Conference on Harmonization (ICH), and local regulations.
3.3. Willing and able to comply with scheduled visits, required study procedures and laboratory tests.
3.4. Women may not be pregnant, lactating or planning pregnancy during the course of the study.
• Includes a negative urine pregnancy test on day of Screening scan prior to injection (DaTSCANTM and/or 18F-AV-133).
• Includes a negative serum pregnancy test prior to Screening scan injection (18F-AV-133 only).
3.5. Women participating in VMAT-2 PET imaging must be of nonchildbearing potential **or** be using a highly effective method of birth control 14 days prior to until at least 24 hours after injection of 18FAV-133).
• Non-child bearing potential is defined as a female that must be either postmenopausal (no menses for at least 12 months prior to Screening) or surgically sterile (bilateral tubal ligation, bilateral oophorectomy or hysterectomy).
• Highly effective method of birth control is defined as practicing at least one of the following: A birth control method that results in a less than 1% per year failure rate when used consistently and correctly, such as oral contraceptives for at least 3 months prior to injection, an intrauterine device (IUD) for at least 2 months prior to injection, or barrier methods, e.g., diaphragm or combination condom and spermicide. Periodic abstinence (e.g. calendar, ovulation, symptothermal, post-ovulation methods) is not acceptable.
**4. Exclusion Criteria (Healthy Control Subjects)**4.1. Current or active clinically significant neurological disorder (in the opinion of the Investigator).
4.2. First degree relative with idiopathic PD (parent, sibling, child).
4.3. MoCA score of 26 or less (i.e., eligible if score is 27 to 30).

4.4. Received any of the following drugs that might interfere with dopamine transporter SPECT imaging: Neuroleptics, metoclopramide, alpha methyldopa, methylphenidate, reserpine, or amphetamine derivative, within 6 months of Screening.
4.5. Subjects participating in VMAT-2 PET imaging have received any of the following medications that might interfere with 18F-AV-133 PET imaging: neuroleptics, metoclopramide, alpha methyldopa, methylphenidate, reserpine, or amphetamine derivative, within 2 weeks prior to the Screening 18F-AV-133 injection.
4.6. Current treatment with anticoagulants (e.g., coumadin, heparin) that might preclude safe completion of the lumbar puncture.
4.7. Condition that precludes the safe performance of routine lumbar puncture, such as prohibitive lumbar spinal disease, bleeding diathesis, or clinically significant coagulopathy or thrombocytopenia.
4.8. Any other medical or psychiatric condition or lab abnormality, which in the opinion of the investigator might preclude participation.
4.9. Use of investigational drugs or devices within 60 days prior to baseline (dietary supplements taken outside of a clinical trial are not exclusionary, e.g., coenzyme Q10).
4.10. Previously obtained MRI scan with evidence of clinically significant neurological disorder (in the opinion of the Investigator).

**The results of primary test statistic threshold testing**

According to the manual of NBS, the test statistics threshold is free. Therefore, we tested a range of primary test statistic thresholds (2.5-3.5, interval 0.1) recommended in the NBS manual to determine the threshold value with the most robust result. We can conclude that the subnetwork containing 10 nodes is robust from our results. To ensure sensitivity, we selected 2.6 as the primary test statistic threshold and the subnetwork including the most edges.

**Table 1. Results of the primary test statistic threshold test**

| primary test statistic threshold | Largest subnetwork components | |
| --- | --- | --- |
|  | edge | node |
| 2.5 | 15 | 15 |
| 2.6 | 12 | 10 |
| 2.7 | 11 | 10 |
| 2.8 | 10 | 10 |
| 2.9 | 9 | 10 |
| 3 | 8 | 9 |
| 3.1 | 2 | 3 |
| 3.2 | 0 | 0 |
| 3.3 | 0 | 0 |
| 3.4 | 0 | 0 |
| 3.5 | 0 | 0 |
